# When Confusion Isn’t Confusion: Anterior Thalamic Infarction Mimicking Encephalitis

**DOI:** 10.7759/cureus.100322

**Published:** 2025-12-29

**Authors:** Zoya Malik, Sadia Faisal, Anika Tabassum

**Affiliations:** 1 Stroke Medicine, Russells Hall Hospital, Dudley, GBR; 2 Acute Medicine, Russells Hall Hospital, Dudley, GBR

**Keywords:** amnesia, anterior thalamic infarct, cognitive dysfunction, confusion, mri brain, ptosis, stroke

## Abstract

A 51-year-old man presented with sudden-onset confusion, impaired recall of familiar names, and isolated left eye ptosis without headache, fever, focal neurological deficits, or loss of consciousness. He was fully conscious on arrival, the Glasgow Coma Scale score was (GCS 15/15) with normal motor and sensory examination, and initial laboratory tests and CT brain imaging were unremarkable apart from low vitamin B12 and vitamin D levels, elevated HbA1c, and raised homocysteine. Empirical acyclovir and Pabrinex were commenced for suspected encephalitis, but although his confusion improved by day four, persistent memory impairment necessitated further evaluation. Differential diagnoses included encephalitis, transient global amnesia (TGA), and stroke; however, TGA was considered less likely due to the prolonged course. Magnetic Resonance Imaging Head later demonstrated a focal T2/FLAIR hyperintensity with restricted diffusion in the left anterior thalamus, confirming an acute thalamic infarct, after which antiplatelet therapy and a statin were initiated, and an outpatient stroke workup was arranged. During admission, he developed stage 3 acute kidney injury secondary to acyclovir, which resolved with treatment cessation and intravenous fluids. At discharge, confusion had resolved, but memory deficits remained, with significant cognitive improvement noted at three-month follow-up despite ongoing reduced concentration. This case highlights the diagnostic challenges of acute cognitive dysfunction without overt focal deficits and underscores the value of early neuroimaging in identifying central causes of confusion.

## Introduction

The thalamus is a key integrative hub for sensory, motor, cognitive, and limbic pathways, and the intricate organization of its nuclei helps explain the wide variation seen in thalamic stroke presentations. Anatomically, the internal medullary lamina divides the thalamus into anterior, medial, and lateral nuclear groups, each of which connects with specific cortical regions and participates in distinct neural circuits [1,2]. The anterior nucleus contributes largely to memory and limbic processing, the mediodorsal nucleus plays a major role in behavior and executive functioning, and the ventral nuclei serve as primary relays for sensory and motor information. Because these nuclei support different functions, lesions in separate thalamic regions can produce diverse and sometimes overlapping clinical syndromes.

This functional complexity parallels the thalamus’s equally intricate vascular supply. Four principal arterial territories arise from branches of the posterior cerebral artery: the tuberothalamic (polar), paramedian, inferolateral (thalamogeniculate), and posterior choroidal arteries [3,4]. The tuberothalamic artery typically supplies the anterior nucleus; the paramedian arteries perfuse the medial thalamus, including the mediodorsal and intralaminar nuclei; the thalamogeniculate arteries supply the ventral posterior and ventral lateral nuclei; and the posterior choroidal arteries feed the pulvinar and geniculate bodies [4,5]. Anatomical variants such as the Artery of Percheron, which can supply bilateral medial thalamic territories, are clinically significant because they may produce symmetric deficits that resemble non-vascular disease processes [6].

The clinical manifestations of thalamic infarction, therefore, depend on the specific vascular territory involved. Infarcts in the anterior (tuberothalamic/polar) territory often result in amnesia, apathy, and confusion - features that may mimic Wernicke encephalopathy, herpes encephalitis, or limbic encephalitis. Historical clues such as nutritional deficiency or alcohol misuse favor Wernicke encephalopathy, preceding febrile or viral symptoms suggest encephalitis, and progressive rather than abrupt onset argues against acute ischemia [2,5,7]. Paramedian infarcts commonly lead to hypersomnolence, vertical gaze disturbances, and behavioral changes, and can be confused with metabolic or toxic encephalopathies or midbrain lesions; sudden onset with ocular motor abnormalities favors medial thalamic ischemia [3,6,8].

Inferolateral (thalamogeniculate) territory strokes typically present with hemisensory deficits or the classic “pure sensory stroke.” However, peripheral neuropathies, cervical radiculopathies, or brainstem disorders can produce similar symptoms; careful history and focused neurological examination (dermatomal pattern, cranial nerve findings) help differentiate [9,10]. Lesions of the posterior choroidal territory often produce visual field defects or visuospatial problems, which can resemble migraine aura or occipital/optic radiation lesions - transient, spreading symptoms point toward migraine, whereas abrupt, fixed deficits suggest ischemia [4,9].

Understanding the structural and vascular anatomy of the thalamus - and recognizing the differential diagnoses associated with each territory - is essential when evaluating suspected thalamic strokes. A detailed, targeted clinical history remains crucial for distinguishing true vascular events from their many mimics.

## Case presentation

A 51-year-old man presented on March 18, 2025, with sudden-onset confusion and left eye ptosis. He was unable to recall the names of colleagues or family members. There was no associated headache, fever, photophobia, loss of consciousness, focal weakness, sensory deficit, or recent travel history. His past medical history included hypertension, for which he was not on regular follow-up.

On examination, he was afebrile with stable vital signs, and his Glasgow Coma Scale (GCS) score was 15/15. Cranial nerve assessment revealed isolated left eye ptosis without diplopia or facial asymmetry. Limb strength, tone, reflexes, and sensation were normal, and no signs of meningeal irritation were noted.

Initial investigations demonstrated vitamin B12 and vitamin D insufficiency, along with elevated HbA1c and homocysteine levels. The remaining laboratory and imaging studies were unremarkable. Given the altered mental state and concern for encephalitis, empirical intravenous acyclovir and Pabrinex were initiated.

On the fourth day of hospitalization, the patient’s confusion gradually improved; however, persistent memory impairment prompted further evaluation by the neurology team. Neurology considered differential diagnoses, including encephalitis, transient global amnesia (TGA), and stroke. TGA was deemed unlikely as the patient’s confusion persisted for more than 24 hours, exceeding the typical duration of a TGA episode. The neurology team advised magnetic resonance imaging (MRI) of the head, lumbar puncture, and a stroke review.

**Figure 1:**
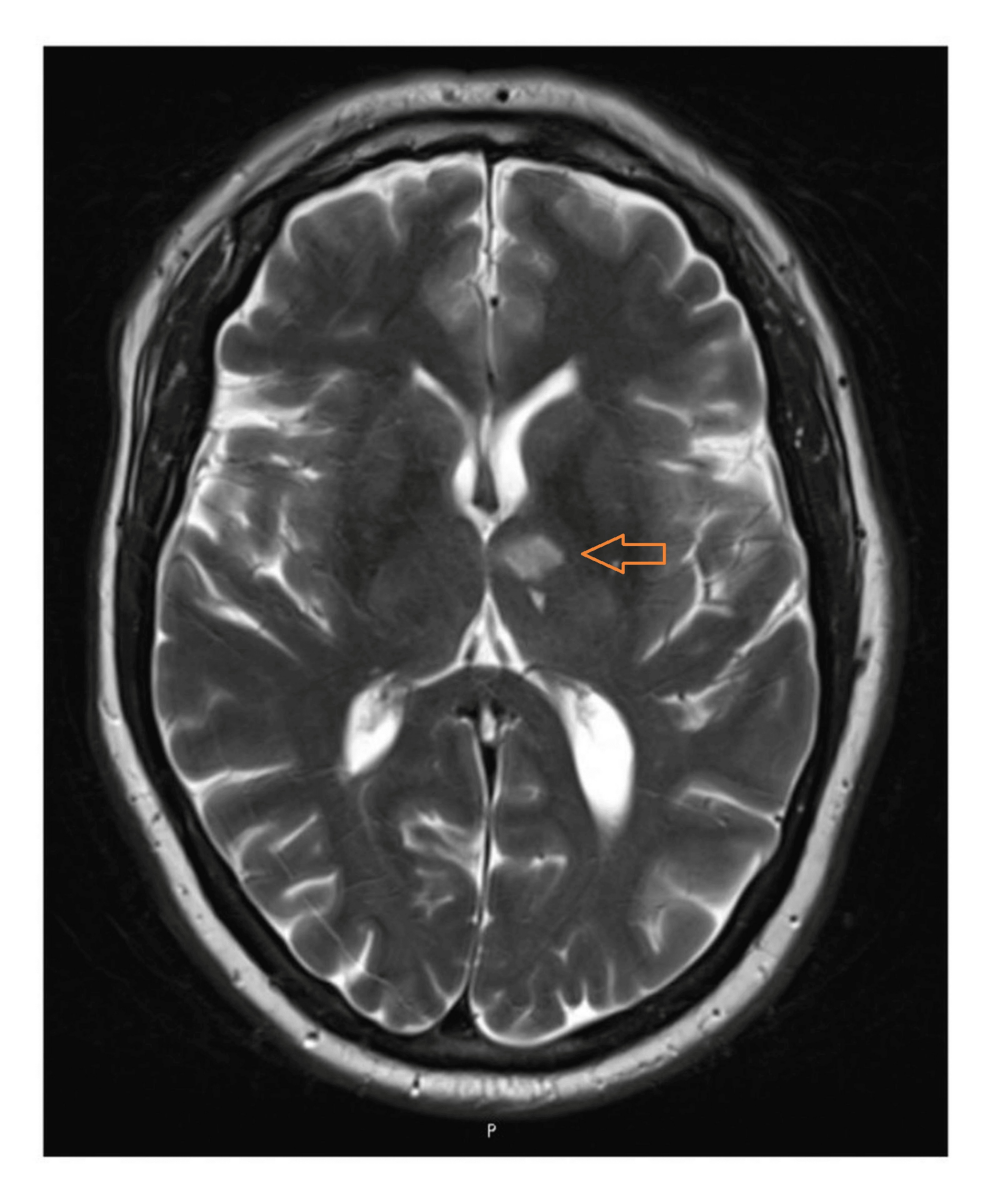
Magnetic Resonance Imaging (MRI) of the head Clinical photograph at presentation showing an anterior thalamic infarct.

An MRI diffusion-weighted imaging (DWI)/FLAIR brain subsequently demonstrated a focal T2/FLAIR hyperintensity with restricted diffusion in the left anterior thalamus, confirming an acute anterior thalamic infarction (Figure 1).

The stroke team was consulted, who confirmed the diagnosis, and dual antiplatelet therapy was initiated with aspirin followed by clopidogrel, along with atorvastatin. A full outpatient stroke workup, including carotid Doppler, electrocardiogram, echocardiography, and young-stroke screening, was scheduled.

During admission, the patient developed acute kidney injury (AKI) stage 3, likely secondary to acyclovir. The drug was discontinued, and he was managed with intravenous fluids, resulting in full renal recovery.

On discharge, neurologically, his memory impairment persisted initially, although he retained intact motor and speech function throughout hospitalization. At a three-month follow-up visit, his confusion had completely resolved, but he continued to report difficulty sustaining prolonged concentration.

## Discussion

Anterior thalamic infarcts are rare and often underrecognized forms of ischemic stroke, most commonly resulting from occlusion of the tuberothalamic artery, a key perforating vessel supplying the anterior thalamic nuclei and associated limbic pathways [5]. These nuclei are integral components of the Papez circuit, a network essential for memory encoding, spatial orientation, and executive functioning [6]. Owing to this unique anatomical role, lesions in the anterior thalamus frequently manifest with acute memory impairment, disorientation, attention deficits, and behavioral changes. Ocular manifestations, including ptosis, may occur when lesions extend to neighboring midbrain structures or involve descending sympathetic pathways [7]. The clinical heterogeneity of such presentations often complicates diagnosis, particularly in the acute setting.

Importantly, anterior thalamic strokes may mimic a range of neurological and psychiatric conditions. Reported mimics include infectious encephalitis, TGA, delirium, and primary psychiatric disorders [8]. The diagnostic challenge is further heightened by the frequent absence of abnormalities on early CT imaging, as small thalamic infarcts may evade detection on non-contrast CT scans. Consequently, clinicians may initially pursue alternative diagnoses, often resulting in unnecessary antimicrobial therapy, delays in stroke management, and potential iatrogenic complications.

In the present case, the patient’s constellation of acute confusion, fluctuating memory impairment, and unilateral ptosis initially led to clinical suspicion of encephalitis or TGA. The normal CT head reinforced these working diagnoses and exemplifies the well-documented diagnostic pitfall associated with isolated thalamic lesions. The subsequent MRI brain with DWI and FLAIR was pivotal, revealing a focal left anterior thalamic infarct and enabling the timely initiation of standardized ischemic stroke management. This aligns with existing literature emphasizing the essential role of MRI in evaluating unexplained acute amnesia and atypical cognitive presentations [8,11,12].

Therapeutically, the patient benefited from antiplatelet therapy, statin optimization, and structured outpatient evaluation for secondary stroke prevention, all consistent with guideline-based management of small vessel and perforator-territory infarction. Although his global confusion resolved, the persistence of reduced concentration at follow-up reflects the subtle long-term cognitive sequelae frequently reported after anterior thalamic injury [9,13]. Such outcomes underscore the critical role of early recognition and targeted rehabilitation in optimizing recovery.

In addition to traditional vascular risk factors (age, diabetes mellitus), elevated homocysteine is increasingly recognized as an independent contributor to cerebrovascular disease. Hyper-homocysteinemia promotes endothelial dysfunction, oxidative stress, vascular inflammation, and a pro-thrombotic state, thereby accelerating atherosclerosis and raising the risk of ischemic stroke. A 2023 meta-analysis found that patients with ischemic stroke had significantly higher homocysteine levels than controls (mean difference + 3.70 µmol/L, 95 % CI 2.42-5.81) [14]. Given that homocysteine levels tend to increase with age and may worsen vascular vulnerability, assessing homocysteine - and correcting modifiable contributors such as B-vitamin deficiencies - could add important prognostic and preventive value in similar cases [15].

Overall, this case reinforces the importance of maintaining a high index of suspicion for thalamic infarction in patients presenting with acute amnesia or confusion, particularly when CT imaging is unremarkable. It highlights the diagnostic value of MRI with DWI and FLAIR and adds to the growing body of evidence characterizing anterior thalamic stroke as a distinct yet often overlooked entity.

## Conclusions

Anterior thalamic infarctions can present with acute confusion, amnesia, and ocular signs such as ptosis, often mimicking encephalitis or TGA. CT imaging may be normal in the acute phase, making MRI with DWI essential for accurate diagnosis. Clinicians should consider thalamic infarcts in patients with these presentations. Early recognition allows for timely stroke-specific management. This approach prevents unnecessary antiviral therapy and reduces diagnostic delays.
